# Editorial: Mechanisms Underlying the Interplay Between Cognition and Motor Control: From Bench to Bedside

**DOI:** 10.3389/fnhum.2022.907278

**Published:** 2022-05-02

**Authors:** Thomas Carsten, Gerard Derosiere, Maximilian J. Wessel, Friedhelm C. Hummel, Julie Duque

**Affiliations:** ^1^CoActions Lab, Institute of Neuroscience, Université catholique Louvain, Brussels, Belgium; ^2^Defitech Chair of Clinical Neuroengineering, Center for Neuroprosthetics (CNP) and Brain Mind Institute (BMI), École polytechnique fédérale de Lausanne (EPFL), Geneva, Switzerland; ^3^Defitech Chair of Clinical Neuroengineering, CNP and BMI, EPFL Valais, Clinique Romande de Réadaptation, Sion, Switzerland; ^4^Department of Neurology, University Hospital, Julius-Maximilians-University, Würzburg, Germany; ^5^Department of Clinical Neurosciences, Geneva University Hospital, Geneva, Switzerland

**Keywords:** movement, cognition, embodied cognition, motor imagery, sensory predictions, impulse control

Movements allow establishing preferred outcomes in the environment. To set movement parameters optimally, a plethora of brain processes spell out the intended course of action, often termed “cognition.” This functional interrelationship between assessment, i.e., cognition, and manipulation, i.e., movement, of the self in the environment suggests that there is crosstalk between “cognitive” and “motor” brain areas. The goal of this Research Topic was to demonstrate and elucidate those mechanisms underlying the interplay between cognition and motor control.

*Is there evidence that cognition and movement are interdependent?* Lunazzi et al. show that the time taken to decide between equivalent candidate movements depends on their duration: Decisions between lengthy reaching movements were faster than those between short movements, indicating that participants aimed to limit the total time needed to obtain rewards. This implies that decisions and movements follow similar overarching goals and hence are subject to common regulatory mechanisms. Ribot et al. demonstrate that well after onset of reaching movements, trajectories are swayed by visual targets representing alternative candidate movements. Hence, decisional processes continuously control movements, so that changes of mind are reflected in deviating movement trajectories. This competition between alternative movements seems to increase GABAergic intracortical inhibition, as indexed by the silent period duration, observed following the motor-evoked potential elicited by Transcranial Magnetic Stimulation. Hence movements may be continuously subject to changes of mind, with GABAergic inhibition potentially serving as gatekeeper. In a patient pilot study, Kim et al. present preliminary evidence that aspects of cognition and motor learning rely on shared neural resources: Motor accuracy and cognitive speed partly suffered more in dual tasks as compared to single tasks for stroke patients in contrast to controls. Re-learning upper limb movements in stroke patients thus puts a strain on cognitive resources, so that daily situations such as answering a question while reaching for an object may be challenging in these patients.

*Which candidate brain structures may promote such interplay?* Grill et al. combined Position Emission Topography and functional Magnetic Resonance Imaging (fMRI) to identify brain regions activated by motor and cognitive aspects of a simple finger tapping task. Several sub regions were identified in the striatum, a subcortical region associated with motor functions, attention and motivation. Some were seemingly more involved in regulating motor aspects of the task, whereas others were sensitive to cognitive aspects. These findings provide further support for the idea that the striatum is neither strictly “cognitive” nor “motoric” but organized along a gradient covering cognition and movement. Boen et al. highlight the functional parcellation of the right Inferior Frontal Gyrus (rIFG) by combining Diffusion Tensor Imaging with behavioral measures. This cortical structure had previously been associated with various cognitive and motor-related functions such as motor inhibition and -imagery, attention and speech. Here, results show that the rIFG is richly connected inter-regionally, with complex cortical and subcortical pathways, some of which likely translate cognitive variables into motor control. More specifically, the dorsal pars opercularis of the rIFG was associated with higher response caution in a stop signal task, but not a simple reaction time task. This suggests that this brain region does not merely assist movement production, but specifically modulates movement when cognitive goals call for response caution.

*Is cognition needed for motor functions?* Thierrien and Wong explore the idea that percepts resulting from simulated movements may play a central role in optimizing behavior over time: Learning to modify movement when environmental or physical states change, i.e., motor adaptation, likely rests on matching predicted and perceived sensory outcomes of own movements. Such sensory prediction errors likely provide important feedback on whether intended movements were successfully executed, but suboptimal in the context of task goals. From this perspective, the line between movement and cognition is blurred in motor adaptation, as matching predicted with actual movement consequences is central to this process. Mathew and Crevecoeur further discuss this idea by arguing that the assumed duality of distinct feedforward and feedback mechanisms in motor adaptation is likely obsolete. Instead, ongoing movement is likely corrected online to match sensory priors set by previous experience, which themselves are optimized over slower time scales to produce better optimized movement. Again, this framework is compatible with the idea that cognition, as assessing the relation between self and environment, and movement, as operation of bringing this relationship to a more favorable state, are inherently intertwined.

*Is movement-related brain activity needed for cognitive functions?* One fascinating implication of this Research Topic is that motor functions may be recruited for cognitive functions. Ridderinkhof et al. investigated the idea that predicting an opponent's shooting direction in football rests on simulating the observed movement as if done by oneself. Utilizing multivoxel pattern analysis on fMRI data, the authors show that such motor-imagery strategies are indeed likely used to predict the shooting direction. As such, brain processes recruited for simulated movement, largely overlapping with those for actual movement, may help inferring movement goals of others, indicating that cognitive functions may rely on motor functions. In a similar fashion, Nalborczyk et al. propose a theory that verbal thought may rely on such overt, “simulated”, movement. In other words, motor-related aspects of thinking such as inner speech, as hallmark of subjective experience underlying cognition, may directly be embedded in motor regions, but inhibited or downregulated to prevent actual speaking. When sensory consequences of speech are simulated instead, such as inner hearing, or when the degree of abstraction is high, verbal thought may rely on motor activity less. When speech and motor inhibition develop during childhood, motor activity may contribute to verbal thought most.

In conclusion, articles collected in this Research Topic suggest that movements are shaped by cognitive functions allowing to reconcile sensory outcomes with cognitive goals. Likewise, cognition may partially rely on “simulated movement” to predict sensory consequences, which are then utilized for abstract operations such as planning, inference or thinking. As such, cognition and movement likely operate as direct functions of each other, rather than in isolation.

## Author Contributions

TC: writing—original draft preparation. GD, MW, FH, and JD: writing—review and editing. All authors contributed to the article and approved the submitted version.

## Conflict of Interest

The authors declare that the research was conducted in the absence of any commercial or financial relationships that could be construed as a potential conflict of interest.

## Publisher's Note

All claims expressed in this article are solely those of the authors and do not necessarily represent those of their affiliated organizations, or those of the publisher, the editors and the reviewers. Any product that may be evaluated in this article, or claim that may be made by its manufacturer, is not guaranteed or endorsed by the publisher.

